# Predictors of irritability in pediatric autistic populations: a scoping review

**DOI:** 10.3389/frcha.2024.1393231

**Published:** 2024-07-23

**Authors:** Sara Alatrash, Tithi Paul, Julia Carbone, Melanie Penner, Atena Roshan Fekr, Azadeh Kushki

**Affiliations:** ^1^ Institute of Biomedical Engineering, University of Toronto, Toronto, ON, Canada; ^2^ Bloorview Research Institute, Holland Bloorview Kids Rehabilitation Hospital, Toronto, ON, Canada; ^3^ Department of Paediatrics, Temerty Faculty of Medicine, University of Toronto, Toronto, ON, Canada; ^4^ KITE Research Institute, Toronto Rehabilitation Institute, University Health Network, Toronto, ON, Canada

**Keywords:** autism, irritability, correlate, predictor, pediatric

## Abstract

**Introduction:**

Autism is characterized by social communication differences and repetitive behaviors, affecting 1%–2% of children. Irritability is a disabling condition affecting 19%–80% of autistic children. While extensive research has focused on interventions to reduce irritability symptoms, the underlying correlates remain poorly understood. To address this, we conducted a scoping review of the literature examining factors contributing to irritability in pediatric autistic populations.

**Methods:**

A literature search of Embase, MEDLINE, PubMed, PsycINFO, and Web of Science was conducted in addition to manually retrieved papers from Google Scholar. Studies underwent title and abstract screening by one reviewer and full-text screening by two reviewers; disagreements were resolved through deliberation. The remaining studies underwent data extraction. The review was conducted using the PRISMA-ScR checklist.

**Results:**

The search yielded 48 studies meeting the inclusion criteria. Correlates of irritability were categorized into eight themes: demographics/environmental, autism features, mental health, language, cognition, and function, neurobiological, physical health, physiological, and multidimensional factors. Findings revealed mixed associations with demographic factors, consistent positive associations with sensory differences and mental health symptoms, and varying associations with cognitive abilities and physical health factors. Neurobiological and physiological correlates were less explored.

**Conclusion:**

This review revealed a significant gap in understanding sociodemographic, phenotypic, and neurobiological and physiological correlates of irritability in autism. There was also a significant gap in understanding the multi-dimensional irritability correlates. Positive associations between irritability and sensory differences and mental health symptoms suggest potential avenues for investigation of non-medication interventions.

## 1 Introduction

Autism (autism spectrum disorder) is defined based on differences in social communication and the presence of intense interests and repetitive behaviors (1)^1^ and impacts 1%–2% of children (2, 3). Autism is more prevalent in males than females (4). Autism often co-occurs with other medical and psychiatric symptoms, such as mental health disorders, sleep difficulties, and gastrointestinal issues, which can contribute to increased experiences of distress and disability (5). Among these, irritability is prevalent and highly disabling and can often lead to a significant decline in quality of life (6).

Irritability is defined as “a feeling state characterized by reduced control over temper which usually results in irascible verbal or behavioral outbursts” (7). This umbrella term often encompasses various moods and behaviors including aggression, intense behavioral responses, and self-injury (8, 9), and is a major reason for referral of children and adolescents for psychiatric assessment and treatment (10, 11). Irritability can be highly disabling and associated with negative short- and long-term outcomes, including anxiety and depression (12), increased risk of suicidal thoughts or actions (11), academic difficulties, and economic hardship (11). Irritability can hinder a child's development, strain relationships, and decrease social and educational functioning (6).

The reported prevalence of irritability in autism is variable but estimates across studies range from 19%–80% (13–15), depending on the sample and measurement tools used. Irritability levels in females were higher than in males suggesting irritability to be more prevalent in autistic females (16). Much of the existing research on irritability has focused on pharmacological and behavioral interventions for reducing irritability symptoms (17). While irritability is the only symptom domain in autism for which medications are approved, these medications can have significant adverse effects (18). An improved understanding of the factors that contribute to irritability can ultimately help inform treatment targets for alternative medications or non-medication interventions. However, very few studies have investigated the correlates of irritability and the association with the core features of autism, co-occurring conditions, or biological mechanisms that may underlie these symptoms (19). These studies have produced mixed findings, and the correlates of irritability symptoms in autism remain poorly understood. To address this gap, we conducted a scoping review of the literature to enhance our understanding of the current state of research on irritability in pediatric autistic populations. Our specific research question for this review was: What is the scope and focus of the literature on factors associated with irritability in children diagnosed with autism?

## 2 Methods

### 2.1 Search strategy

Five online databases were searched targeting correlates and predictors of irritability in autism. The databases were Embase, MEDLINE, PubMed, PsycINFO, Web of Science in addition to manually retrieving papers from Google Scholar. Subject heading and key words were tailored to search for the population of interest (autism) and concept (irritability) using the Boolean operator “AND”. These terms were entered and truncated or broadened to ensure widespread coverage. For example, “irrita*” captures terms such as “irritability”, “irritable”, and “irritate”; while “autis*” captures terms such as “autism” and “autistic”. No date constraints were applied to the search in any of the databases. The search was completed in September 2023. The review was conducted using the PRISMA-ScR (Preferred Reporting Items for Systematic reviews and Meta-Analyses extension for Scoping Reviews) checklist.

### 2.2 Inclusion and exclusion criteria

Inclusion criteria for the review were: (1) the study examined correlates/predictors of irritability, (2) study sample included individuals with an autism diagnosis, (3) study sample includes pediatric population (children from birth to 18 years of age), and (4) manuscript was written in English. Studies focusing on interventions and treatments, literature reviews, theses, conference abstracts, and books were excluded.

### 2.3 Data extraction

All articles were imported to Covidence for screening, review, risk of bias assessment, and extraction by the authors (screening and extraction: SA; full-text review: SA, TP). For full-text review, each study was assessed by two reviewers, and disagreements were resolved through deliberation. Inter-rater reliability was determined on a subset of 15 articles, achieving a Cohen's kappa of approximately 0.73, which indicates substantial agreement beyond chance between the two raters (20).

Data were extracted using custom extraction templates on Covidence. The extracted data included the following: title, author, year, country, aim, and sample characteristics [total sample size, sex (biological), gender (identity), age, social/economic status, and race/ethnicity]. Other data extracted included predictors/correlates of irritability, analysis methods, measures, informants, and key findings. During the data extraction, one reviewer extracted the data, and a second reviewer cross-checked the results. An adapted version of the Cochrane template was used for the risk of bias assessment (see Supplementary Table S1).

## 3 Results

**Figure 1:**
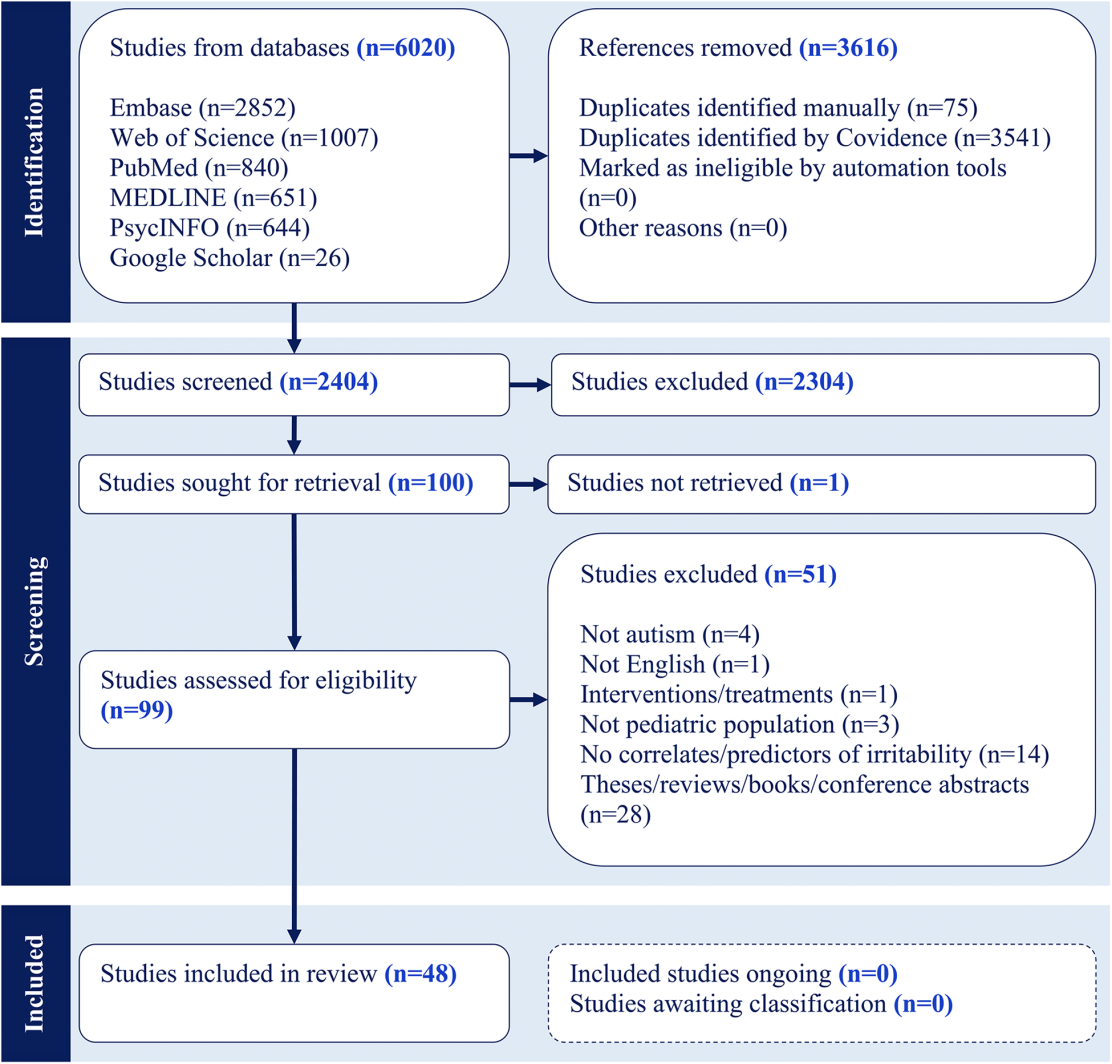
Scoping review PRISMA.

The literature search process is presented in Figure 1. Of the 6,020 articles identified by the search, 3,616 (60%) were identified as duplicates. The first screening of titles and abstracts eliminated 2,304 articles based on the inclusion criteria, leaving 100 articles for the full-text review. Following the full-text review, 51 articles were eliminated according to the exclusion criteria. The full text of one article was not retrieved. The final review included 48 studies summarized in Table 1 (see Supplementary Table S2 for more details on these studies).

**Table 1:** Summary of characteristics of the reviewed studies.

Study	*n* (% male)	Age (years) Range Mean (SD)	Key findings
Anderson et al. (21)	65 (90%)	9–18 T1: 9.7 (2.22) T2: 18.1 (1.86)	Irritability negatively associated with age, no longer significant after controlling for IQ.
Baeza-Velasco et al. (22)	152 (82%)	13–17 4.9 (1.60)	Irritability significantly correlated with hyperactivity, stereotypy, and lethargy domains of ABC.
Bangerter et al. (23)	144 (78%)	≥6 14.6 (7.83)	Irritability positively associated with caregiver-reported sleep problems, but not actigraphy measures.
Bitsika et al. (24)	150 (100%)	6–18 11.2 (3.33)	Irritability negatively associated with age, and positively with anxiety.
Bitsika et al. (25)	150 (100%)	6–18 11.2 (3.33)	Irritability negatively associated with age, and positively with depression symptoms.
Brenner et al. (26)	350 (79%)	4–21 12.9 (3.30)	Irritability positively associated with history of abuse.
Carpenter et al. (27)	185 (81%)	3–7 4.8 (1.20)	Irritability positively associated with social adaptive behaviors.
Carter Leno et al. (28)	52 (63%)	13–17 15.4 (1.10)	Irritability not significantly correlated with autism features, age, sex, or IQ, but positively correlated with ODD symptoms, and a flatter slope of heart rate.
Chaidez et al. (29)	499 (86%)	2–5	Irritability positively associated with gastrointestinal symptoms.
Curran et al. (30)	30 (80%)	2–18 7.4 (3.30)	Irritability not associated with fever.
Dellapiazza et al. (31)	197 (83%)	3–10 5.7 (2.20)	Irritability positively associated with sensory differences, and negatively with IQ.
Dellapiazza et al. (32)	51 (85%)	3–10 5.5 (2.10)	Irritability positively associated with sensory differences.
Estes et al. (33)	74 (82%)	6 6.1 (0.23)	Irritability positively associated with non-verbal IQ and communication abilities.
Ferguson et al. (34)	120 (90%)	6–18 11.8 (3.80)	Irritability negatively associated with cytokines.
Ferguson et al. (35)	120 (90%)	6–18 11.8 (3.80)	Irritability positively associated with gastrointestinal symptoms.
Flowers et al. (36)	145 (79%)	9–21 16.0 (3.26)	Irritability positively correlated with sensory differences, stereotypies, self-injurious behaviors, and aggression, but not with age, sex, adaptive behavior/skills.
Fok et al. (37)	1,937 (87%)	6–18 10.1 (2.96)	Irritability positively associated with externalizing and internalizing behaviours and negatively associated with verbal ability, but not after controlling for non-verbal IQ.
Frazier et al. (38)	2,418 (87%)	4–18 F: 9.3 (3.76) M: 9.0 (3.56)	Irritability higher in females than males, negatively correlated with age, and not associated with IQ.
Frye et al. (39)	87 (80%)	6.8 (3.08)	Irritability significantly associated with thyroid function.
Gabriels et al. (40)	14 (71%)	10.6 (7.00)	Irritability positively associated with non-verbal IQ. Irritability positively associated with repetitive behavior, but not after controlling for non-verbal IQ.
Gotham et al. (41)	1,429 (86%)	5–18 10.2 (3.08)	Anxiety significantly predicted irritability.
Graziosi et al. (16)	457 (79%)	4–20 13.0 (3.33)	Irritability negatively associated with adaptive behaviors and age, and female sex. Irritability not associated with non-verbal IQ.
Griffin et al. (42)	75 (87%)	7–12 7.8 (2.61)	Irritability positively associated with sensory differences and caregiver strain.
Gundogdu et al. (43)	46 (83%)	3–9 5.3 (2.17)	Irritability positively associated with sensory differences, autism features, and Otoacoustic Emission test failure.
Hartley-McAndrew et al. (44)	21 (81%)	7–23 9.5	Irritability not significant correlated with seizures.
Henry et al. (45)	123 (83%)	2–18	Irritability positively associated with family history of mood disorders.
Hirota et al. (46)	2,612 (88%)	4–18	Irritability links aggressive behaviours to other psychopathological symptoms in the network.
Johnson et al. (47)	177 (88%)	3–7 4.7 (1.14)	Irritability significantly positively associated with sleep problems.
Kalvin et al. (9)	81 (78%)	8–16 G1: 12.2 (1.75) G2: 12.7 (2.06) G3: 12.5 (2.19)	Irritability positively associated with anxiety, noncompliance, and repetitive and restricted behavior. No association with IQ, age, and sex.
Kryza-Lacombe et al. (48)	47 (83%)	8.3–19.2 13.9 (2.35)	Irritability levels negatively associated with activation in the left middle frontal gyrus and left inferior frontal gyrus in response to both fearful and happy faces, as well as altered connectivity between the right amygdala and left superior frontal gyrus.
Lee et al. (49)	345 (79%)	6–18 14.3 (2.70)	Irritability not significantly associated with executive dysfunctions.
Lundwall et al. (50)	45 (100%)	3–13	Irritability negatively associated with brainstem volume.
Martinez-Gonzalez et al. (51)	62 (81%)	G1: 10.9 (5.46) G2: 12.4 (7.75)	Irritability not statistically significantly different before and during COVID-19 confinement.
Mayes et al. (52)	1,436 (79%)	2–17 6.6 (3.30)	Irritability not significantly different between females and males.
Mayes et al. (53)	1,436 (79%)	2–17 6.6 (3.30)	Autistic children with IQ less than 70 had significantly less irritability.
Mazurek et al. (54)	81 (86%)	3.6–19.6 10.3 (3.80)	Irritability higher in females than males, but not significantly associated with race, age, bedtime resistance, sleep onset delay, or sleep disordered breathing. Irritability significantly positively correlated with physical aggression, inattention, hyperactivity, sleep anxiety, sleep duration, night wakings, daytime sleepiness, and parasomnias.
Mikita et al. (55)	47 (100%)	10–16 12.8 (2.00)	Higher irritability associated with dampened physiological response to stress (muted cortisol response)
Molcho-Haimovich et al. (56)	237 (77%)	1.4–8.7 4.4 (1.49)	Irritability positively associated with sensory differences and sleep disturbances, but not sex.
Nelson et al. (57)	30 (90%)	9–21 14.9 (2.99)	Irritability negatively associated with family functioning, daily activity, and emotional domains of PedsQL. Not associated with physical, social, cognitive, communication, worry, and family relations scores on the PedsQL.
Neuhaus et al. (58)	2,079 (87%)	4–18 10.3 (3.12)	Irritability significantly positively associated with social skill difficulties and female sex, and negatively with adaptive functioning.
Ogur et al. (59)	12 (100%)	3–15	Irritability negatively associated with fractional anisotropy in the left frontoparietal anterior limb of the right internal capsule and left middle cerebellar peduncle.
Rosen et al. (60)	165 (79%)	9–18 T1: 10.0 (0.89) T2: 19.0 (1.20)	Irritability not associated with family composition (siblings).
Sannar et al. (61)	106 (76%)	4–20 12.9 (3.40)	Irritability positively associated with sleep difficulties. Irritability not significantly correlated with number of minutes slept or total number of awakenings.
Turkoglu et al. (62)	46 (83%)	4–17 7.9	Irritability significantly higher during the COVID-19 confinement period.
Valicenti-McDermott et al. (63)	50 (94%)	2–18 8.8 (3.00)	Irritability not significantly associated with sleep difficulties.
Viscidi et al. (64)	2,645 (87%)	4–18 9.0 (3.60)	Irritability significantly higher in females than males, positively associated with seizures.
Williams et al. (65)	346 (79%)	4–21 12.9 (3.30)	Irritability positively associated with verbal ability.
Yavuz-Kodat et al. (66)	52 (79%)	3–10 5.4 (1.50)	Irritability positively associated with sleep difficulties.

**Figure 2:**
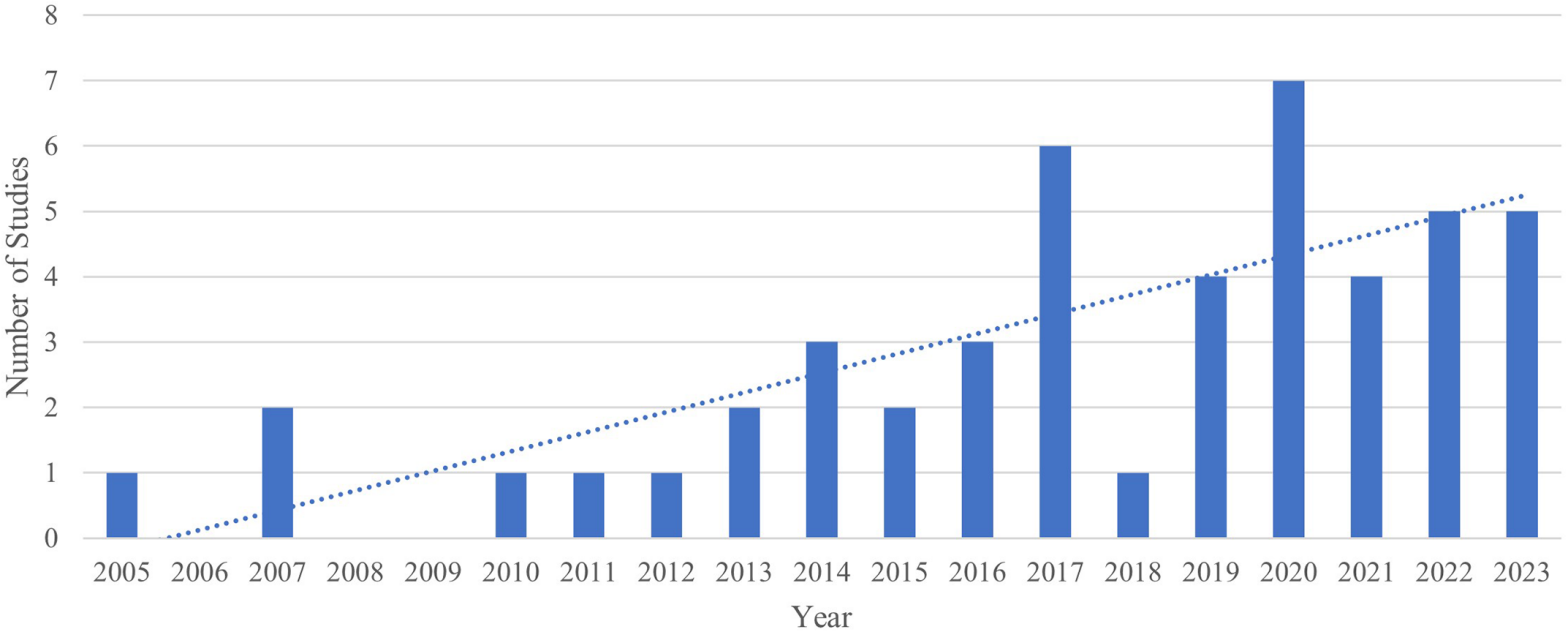
Number of studies across different years.

There was a predominance of male participants in all the studies (85%). Most studies were completed in the United States of America (*n* = 33), followed by France (*n* = 4), Australia (*n* = 3), and Turkey (*n* = 3). None of the 48 studies reported gender identity, 27 reported race/ethnicity, and 18 reported on social/economic status. The irritability subscale of the Aberrant Behavior Checklist ABC-I was the most common measure of irritability (*n* = 39), followed by the Affective Reactivity Index ARI (*n* = 5). Figure 2 shows the number of reviewed studies across years.

Among the studies reviewed, most (*n* = 36) were found to have a low risk of bias. Only eleven studies had a medium risk of bias, and one study had a high risk of bias. The most common sources of bias were related to sample selection and description, the description of statistical methods, and the reporting of statistical results.

The results are presented using a narrative approach. We grouped the predictors examined in the reviewed literature into 8 themes defined as follows (Table 2):

1. Demographics and environmental factors defined as variables that describe personal characteristics and external influences, including family history.
2. Autism features defined as variables that quantify core autism features, including social communication differences, intense interests, repetitive behaviors, and sensory differences.
3. Mental health factors defined as variables related to mental and emotional well-being, including internalizing and externalizing behaviors.
4. Language, Cognition, and Function factors defined as predictors that describe language and cognitive abilities include expressive and receptive language, Intelligence Quotient (IQ), and functioning.
5. Neurobiological factors defined as predictors that characterize brain structure, function, connectivity, and neurochemistry.
6. Physical health factors defined as predictors related to general well-being, and medical conditions impacting physical health.
7. Physiological factors defined as predictors that quantify the function of body organs and other physiological processes such as heart rate, heart rate variability, and skin conductance.
8. Multidimensional factors refer to the category of studies that considered multi-dimensional interactions among variables from the above categories.

**Table 2:** Synthesis of review results.

Category	#	Fields	Main findings
Demographics/environmental	19	Demographics, COVID-19 confinement, family history, and abuse.	Age and sex show varied correlation with irritability. No significant correlation between irritability and race or family composition. Family history of mood disorders and reported abuse associated with irritability. Increased irritability observed during COVID-19 confinement. Irritability predicted caregiver strain.
Autism features	12	Social behavior, restrictive interests, repetitive behaviors, and sensory differences.	Atypical sensory processing, repetitive behavior, and restricted behavior linked to irritability. Irritability not significantly correlated with autism severity.
Mental health	10	Emotion regulation and internalizing and externalizing behaviors.	Generalized anxiety disorder (GAD) positively associated with irritability. Irritability significantly predicted GAD, depression, and self-injurious behavior frequency and severity. Irritability correlates with anxiety, noncompliance, depression, ODD symptoms, self-injurious behavior, aggression, inattention, hyperactivity, sleep anxiety, parasomnias.
Language, cognition, and function	13	Language, intellectual functioning, executive and adaptive functioning.	IQ below 70 associated with significantly less irritability. Irritability decreases as best estimate IQ increases. Low intellectual functioning at age 6 linked to higher irritability by age 9. Verbal ability and adapting/coping are significant predictors of irritability. Higher irritability associated with differences in social adaptive behavior.
Neurobiological	3	Brain structure, function, and neurochemistry.	Negative association between irritability and white matter connectivity. Altered amygdala connectivity linked to increased irritability. Smaller brainstem volumes associated with higher odds of high irritability.
Physical health	14	Gastrointestinal, sleep, hyperthyroidism, fever, and seizures.	Gastrointestinal symptoms significantly linked to higher irritability. Thyroid dysfunction associated with changes in irritability scores. No connection found between fever and irritability severity. Inner ear response to sound correlated with irritability. Sleep problems contribute to increased irritability.
Physiological	2	Heart rate, heart rate variability, cortisol, and skin conductance.	Higher irritability initially showed a dampened physiological response to frustration, but this association became non-significant after considering other factors. Higher levels of irritability are linked to a dampened physiological response, including heart rate and cortisol levels, during stress tasks.
Multidimensional	3	Autism features, physical health, demographics/environmental, and mental health.	Irritability has stronger connections with symptoms than aggression. Linear model ties sex, bedtime resistance, sleep duration, anxiety, and night wakings to irritability/hostility. Irritability negatively correlates with family functioning, daily activity, emotional scores on PedsQL, no correlation in other domains.

**Figure 3:**
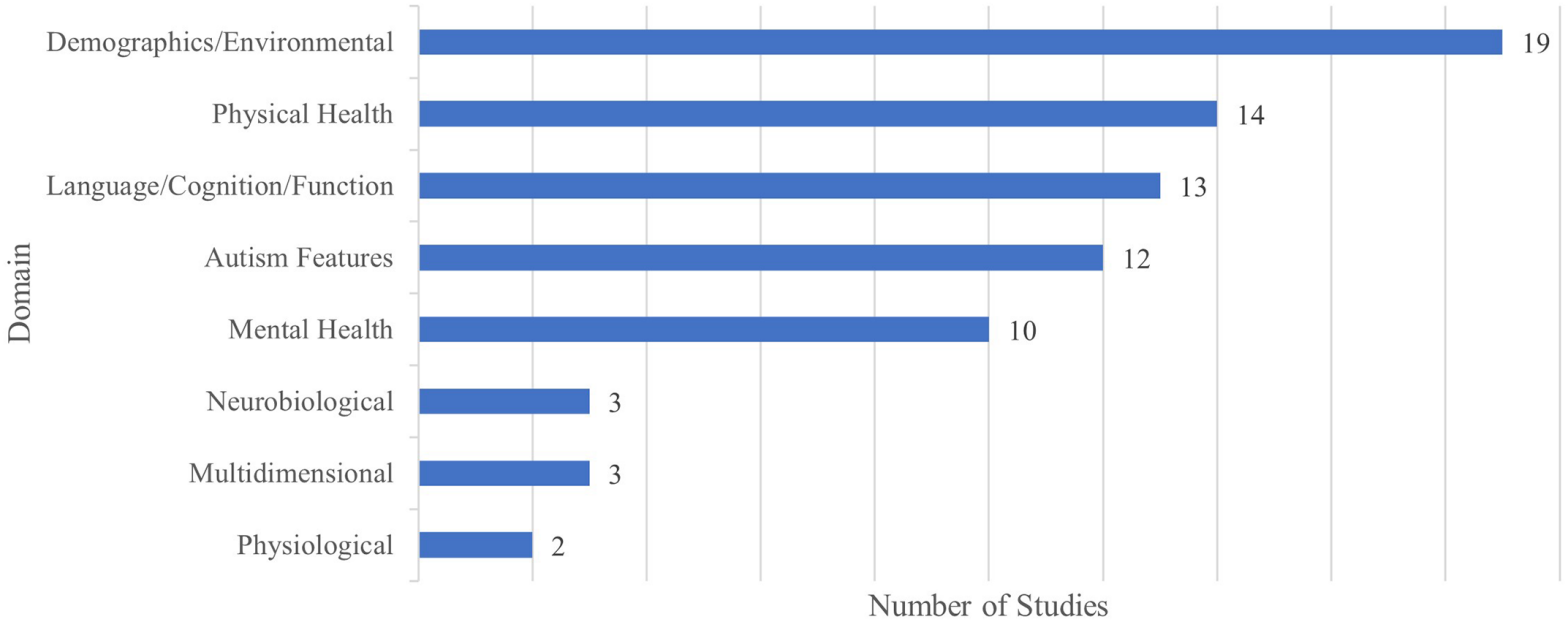
Number of studies under each domain.

Figure 3 shows the number of reviewed studies under each domain.

### 3.1 Demographics and environmental factors

Nineteen studies examined demographic and environmental factors as predictors of irritability, including age, sex, race, family composition, and family history of mental health challenges.

The most studied demographic factors were sex and age; however, the findings related to the association between these variables and irritability were mixed. Of the ten studies examining the association between sex and irritability, five studies found higher levels of irritability in females compared to males (16, 38, 54, 58, 64), while five found no significant sex differences in irritability levels (9, 28, 36, 52, 56). Eight studies examined the effect of age on irritability, with four studies reporting a significant negative association between age and irritability (16, 21, 24, 25), and four reporting no significant associations between the two variables (9, 28, 36, 54).

Only one study examined the effect of race on irritability (54), reporting no statistically significant association between the two variables. Family composition was also not found to be significantly associated with irritability (54, 60). Family history of mood disorders and caregiver strain were associated with increased irritability symptoms in one study (42, 45).

Other studies reported higher levels of irritability in those with a history of abuse (26), as well as during COVID-19 confinement period (51, 62).

### 3.2 Autism features

Twelve studies investigated autism features as predictors of irritability. Among these, six studies examined the associations between irritability and differences in sensory processing, consistently suggesting a significant positive association between the two (31, 32, 36, 42, 43, 56). Two studies examined the association between irritability and the overall intensity of autism features (28, 58, 62); one did not find a statistically significant association between the variables (28), while the other reported a positive association (58). Functioning in the social domain was negatively associated with irritability in two studies (27, 58). Positive associations were also reported between irritability and repetitive behaviors in two studies (9, 40), although the association was no longer significant after correcting for non-verbal IQ (40).

### 3.3 Mental health factors

Ten studies examined mental health symptoms as predictors of irritability. Overall, the results were consistent with a positive association between irritability and mental health challenges including anxiety (9, 24, 41, 55), depression (25), self-injury (36), oppositional defiant disorder (ODD) symptoms (28), hyperactivity, stereotypy, and lethargy (22), symptoms of attention deficit hyperactivity disorder (ADHD) (54), and internalizing and externalizing symptoms (37).

### 3.4 Language, cognition, and function factors

Thirteen studies investigated the association between irritability and language and cognitive abilities. Of these, seven focused on cognitive ability, reporting mixed results. In particular, one study found autistic children with IQ below 70 showed significantly less irritability (53), three studies found no association between IQ and irritability (9, 16, 28), and three found a negative association between irritability and IQ (31, 33, 40).

The results for the association of irritability and language were also mixed. Of the three papers reporting on this, one study found a positive association between irritability and verbal ability (65), and two studies found no significant association between the two domains after adjusting for non-verbal IQ (21, 37).

Three studies examined the association between adaptive functioning and irritability. No significant association was found in one study (36), while a negative association was reported in two other studies (16, 58).

Only one study examined the association between irritability and executive dysfunctions, reporting no significant association between the two domains (49).

### 3.5 Neurobiological factors

Three studies investigated neurobiological correlates of irritability in autism (48, 50, 59). One study found a negative association between irritability and fractional anisotropy in the left frontoparietal anterior limb of the right internal capsule and left middle cerebellar peduncle (59). One study found that increased irritability levels in youths are associated with decreased activation in the left middle frontal gyrus and left inferior frontal gyrus in response to both fearful and happy faces, as well as altered connectivity between the right amygdala and left superior frontal gyrus during the perception of fearful and sad faces (48). Brainstem volumes were found to be negatively associated with irritability (50).

### 3.6 Physical health factors

Fourteen studies explored physical health factors as correlates of irritability including sleep (23, 47, 54, 56, 61, 63, 66), gastrointestinal distress (29, 34, 35), hyperthyroidism (39), fever (30), and seizures (44, 64).

Seven studies reported on the association between irritability and sleep difficulties, consistently reporting positive associations between the two domains (23, 47, 54, 56, 61, 63, 66). Irritability and gastrointestinal problems were also found to be positively associated across three reviewed studies (29, 34, 35). Only two studies examined the association between seizures and irritability, reporting positive (64) or no associations (44).

Other physical health variables examined in the reviewed literature included thyroid function (significantly associated with irritability) (39) and fever (no association with irritability) (30).

### 3.7 Physiological factors

Of the reviewed literature, two studies found that higher levels of irritability were associated with a dampened physiological response to frustration (28) and stress (55), including a decreased heart rate change (28) and a muted cortisol response (55); However, the association between heat rate change lost significance when controlling for IQ, autism features, and medication use as covariates (28).

### 3.8 Multidimensional factors

Three studies investigated multi-dimensional predictors of irritability (46, 54, 57). In one study, irritability was predicted by the combined factors of sex and sleep difficulties (bedtime resistance, sleep duration, sleep anxiety, and night wakings) (54). In another study (57), irritability was negatively associated with the total score and subdomain scores on the family functioning, daily activity, and emotional domains of the Pediatric Quality of Life Inventory (PedsQL), while no associations were observed with physical, social, cognitive, communication, worry, and family relations scores on the PedsQL (57).

One study found that irritability may be a bridge linking aggressive behaviours and other symptoms (46). The study treated irritability and aggression as one construct, but irritability's stronger connections to other behaviors suggest they might be distinct concepts.

## 4 Discussion

We conducted this scoping review to examine the existing literature on the correlates of irritability in autism. Our review identified 48 published studies that investigated the correlates in eight categories of demographic/environmental factors, autism features, mental health, language and cognitive abilities, neurobiological features, physical health, and multidimensional analysis. Overall, the review identified significant gaps in understanding irritability correlates in autism. This gap was particularly evident in understanding the association of irritability with demographic variables, core and co-occurring features of autism, and neurobiological and physiological factors.

In terms of demographic correlates of irritability, the reviewed literature was focused on age and sex, reporting highly mixed results. This likely reflects the heterogeneity in study samples, differences in the definition of irritability and analytical approaches, and the complexity of the association of irritability with sex and age. For example, sex and gender are suggested to influence, as mediators or moderators, the phenotypic expression of core and co-occurring features and their developmental trajectories in autism (67), which can in turn be associated with irritability levels. At the same time, developmental trajectories of autism are highly heterogeneous and can be impacted by both biological and environmental factors (67). This suggests that the investigation of sex and age differences cannot happen in silo and should consider the interactions of these factors with phenotypic and environmental variables.

Our review revealed significant gaps in the reporting and investigation of gender identity as well as other demographic factors such as race, socioeconomic status, and other dimensions of identity. This is a critical avenue for future research as these variables contribute to disparities in access to resources and services that can significantly impact the experiences and expressions of distress (68, 69).

In terms of autism features, the most consistent findings suggested a positive association between sensory differences and irritability. This is not surprising given the significant negative impact of adverse sensory experiences in autistic individuals (70, 71). This highlights the importance of considering sensory needs and environmental accommodations in management of irritability symptoms in autism. At the time, future research is needed to examine the association of irritability with individual differences in sensory experiences and whether specific sensory profiles may enhance the likelihood or intensity of irritability symptoms. Our review revealed very few studies examining the association of irritability with other core features of autism (social communication, repetitive behaviors/intense interests).

There is a consistent positive association between irritability and various mental health challenges, including anxiety, depression, self-injury, symptoms of ODD, and symptoms of ADHD, which highlights the complexity of emotional well-being and behavioral outcomes in the autistic population. These findings suggest that autistic individuals who experience heightened levels of anxiety or depressive symptoms, as well as those with symptoms of ADHD, may be at increased likelihood for exhibiting irritability. Moreover, the correlation between irritability and hyperactivity, stereotypy, lethargy, and externalizing symptoms further emphasizes the diverse pathways through which emotional dysregulation manifests. These results must be interpreted cautiously in the context of the significant construct overlap between irritability and mental health symptoms (e.g., externalizing behaviours). Future studies are also needed to examine the directionality of this association to inform potential intervention targets.

Surprisingly, the results of studies examining the association of cognitive and language abilities with irritability were highly mixed, suggesting positive, null, and negative associations. This may be a result of differences in the ranges of IQ considered, thresholds used for IQ dichotomization, or variability in the analytic approaches, including covariates used. Nevertheless, the lack of consistent findings in this area is particularly concerning given that antipsychotic medications, approved for treatment of irritability in autism, are prescribed at higher rates in individuals with intellectual disability (ID) compared to those without, and were the most commonly used medication class in this group (72). This highlights the significant need for future studies clarifying the construct and assessment of irritability in those with lower scores and IQ tests, as well as the interaction of cognitive ability with other factors that may impact the perceived levels of irritability (e.g., communication ability, access to other interventions).

In the domain of physical health, sleep difficulties and gastrointestinal distress were consistently associated with increased irritability. Again, future work is needed to elucidate the directionality of these associations. Other than sleep, very few physical health domains were examined.

Our review also found very few studies examining the neurobiological and physiological correlates of irritability in autism. Further investigation of these factors is critically needed to enhance our understanding of the mechanistic process underlying irritability, potentially informing new intervention targets. A promising avenue in this direction is improved characterization of arousal modulation and its association with irritability (both non-medication and medication approaches exist for arousal modification). While there are significant gaps in understanding the association of irritability and arousal modulation, there is a large body of evidence suggesting atypical arousal processes in autism, including physiological hyperarousal and dampened reactivity to stressful tasks (73–75).

Finally, one of the most critical limitations of the existing literature is that correlates of irritability are almost always considered in silos. However, many of the predictors reviewed above can be highly correlated (for example, sex and symptom domains, mental health and core symptoms, IQ, and mental health). Studying these predictors in silos makes it very challenging to identify and isolate factors that may drive irritability. Multi-dimensional investigations of irritability are critically needed to address this gap.

Future research should further investigate the predictors and physiological associations of irritability in autistic individuals, particularly the multi-dimensional associations between irritability and demographics, autism features, sensory processing, emotion regulation, and internalizing and externalizing problems. Understanding these associations can inform targeted intervention strategies and highlight priority areas for support. Additionally, examining the link between physiological arousal and irritability can provide insights into underlying mechanisms, again informing targeted interventions. Understanding the predictors of irritability in pediatric autistic populations holds significant implications for clinical practice. It can facilitate the early identification of individuals with a higher likelihood of having irritability, enable the tailoring of interventions based on specific predictors identified in the study, and enhance overall care strategies for children experiencing irritability. These insights have the potential to improve diagnostic accuracy, optimize treatment effectiveness, and ultimately enhance patient outcomes in clinical settings.

This review had various strengths. It synthesizes the current body of literature to identify gaps and highlight areas for future research on irritability in autism. A notable strength is its adherence to standard review methodologies, as demonstrated by the use of two independent reviewers who examined each citation and article and met regularly to resolve any conflicts. One notable limitation of this review is its exclusive focus on studies conducted in English, with the majority of reviewed studies conducted in the United States. This limits the generalizability of results to diverse cultural contexts where the presentation and interpretation of irritability among autistic individuals may vary.

## 5 Conclusion

This review revealed a significant paucity of literature examining the sociodemographic, phenotypic, and neurobiological and physiological correlates of irritability in autism. There was also a significant gap in understanding the multi-dimensional irritability correlates. Consistent positive associations were found between irritability and sensory differences and mental health symptoms, suggesting potential avenues for investigation of non-medication interventions.

## Data Availability

The original contributions presented in the study are included in the article/Supplementary Material, further inquiries can be directed to the corresponding author.

## Footnotes

^1^We recognize that there are differences in language conventions for referring to the autistic identity. In this paper, identity-first language (“autistic children”) will be used.
