# Supplementary material for: Predictors of irritability in pediatric autistic populations: a scoping review
Source: Front Child Adolesc Psychiatry. 2024 Jul 23;3:1393231. doi: 10.3389/frcha.2024.1393231 (PMC11747912; doi:10.3389/frcha.2024.1393231)
Supplement: Supplementary file 1 [file Table1.docx]

Supplementary Material

# Supplementary Tables

**Supplementary Table 1** Risk of Bias Questionnaire Adapted from the Cochrane Template.

| **Question** | **Description** |
| --- | --- |
| **Risk of bias in the selection process for the target population (Was the selection process appropriate)?** | High bias: Participants differ from the population of interest. For example: the study recruits children of mostly higher socioeconomic status.  Participants should be described in detail (i.e., sociodemographic factors at least mentioning one race/ethnicity or socioeconomic)  1. High 2. Low 3. Some Concern |
| **Risk of bias in describing target population (Was the target population clearly defined)?** | High bias: Inclusion and exclusion criteria are not specified clearly. For example: the study says, "Participants with autism-like symptoms were recruited" and provides no further detail.  Sufficient detail should be provided about participants.  1. High 2. Low 3. Some Concern |
| **Risk of bias in outcome statistics reporting (Is it clearly stated?)** | High bias: Only a subset of original outcomes measured/analyzed are reported. For example, results that are not significant are deliberately not included in the results.  Outcomes should be comprehensively reported, and no omission of outcomes mentioned, or underreporting should take place.  1. High 2. Low 3. Some Concern |
| **Risk of bias in statistical methods (Are the statistical methods clearly described)** | High bias: The statistical approach was not appropriate and/or the statistical methods were poorly described. For example: A study using only correlations to determine the association with an outcome predictor or if statistical analysis is not described in detail. The methods section should be detailed and comprehensive for the reader.  1. High 2. Low 3. Some Concern |
| **Other Bias** | State any important concerns about bias not addressed in the other domains in the tool. If questions/entries were pre-specified in the review’s protocol, responses should be provided for each question/entry. |
