# Supplementary material for: Predictors of irritability in pediatric autistic populations: a scoping review
Source: Front Child Adolesc Psychiatry. 2024 Jul 23;3:1393231. doi: 10.3389/frcha.2024.1393231 (PMC11747912; doi:10.3389/frcha.2024.1393231)
Supplement: Supplementary file 2 [file Table2.docx]

Supplementary Material

# Supplementary Tables

**Supplementary Table 2** Detailed data extracted from studies included in the scoping review.

| **Study ID** | **Domain / Level of Bias** | **Country of Origin** | **n (% male)** | **Age (Year): range**  **mean (SD)** | **Gender Reported** | **Race/Ethnicity Reported** | **Social/Economic Reported: Details** | **Measure of Autism** | **Measure of Irritability (Informant)** | **Other measures** | **Title** | **Aim of study** | **Analysis Method** | **Key Findings** |
| --- | --- | --- | --- | --- | --- | --- | --- | --- | --- | --- | --- | --- | --- | --- |
| **Anderson et al. (2011) (29)** | a, d / **L** | US | 65 (90%) | 9-18  T1: 9.7 (2.22), T2: 18.1 (1.86) | No | Yes | Yes: Maternal education | ADI-R, ADOS | ABC (Parent/Caregiver) | WISC-3, DAS, Mullen Scales of Early Learning (Mullen) | Changes in Maladaptive Behaviors from Mid-Childhood to Young Adulthood in Autism Spectrum Disorder. | To examine trajectories of change in symptoms of irritability, hyperactivity, and social withdrawal, as well as predictors of such behaviors for youths with ASD and a comparison group with non-spectrum developmental delays. | Growth Curve Analysis and Standardized Mean Difference (SMD). | Irritability negatively associated with age, no longer significant after controlling for IQ. |
| **Baeza-Velasco et al. (2014) (45)** | c / **L** | France | 152 (82%) | 13-17  4.9 (1.60) | No | No | Yes: Parental SES | CARS | ABC (Parent/Caregiver) |  | Are Aberrant Behavioral patterns associated with the adaptive behavior trajectories of teenagers with Autism Spectrum Disorders? | To identify Aberrant Behavioral patterns in adolescents with autism spectrum disorders and to examine if these patterns are associated with their adaptive behavior trajectories. | Spearman correlations and Hierarchical cluster analysis (HCA). | Irritability significantly correlated with hyperactivity, stereotypy, and lethargy domains of ABC. |
| **Bangerter et al. (2020) (56)** | F / **S** | US | 144 (78%) | ≥6  14.6 (7.83) | No | No | No | ABI, SRS-2 | ABC (Parent/Caregiver) |  | Relationship Between Sleep and Behavior in Autism Spectrum Disorder: Exploring the Impact of Sleep Variability. | To examine the relationship between sleep and other caregiver-reported behaviors in children and adults with autism spectrum disorder. | Spearman correlation. | Irritability positively associated with caregiver-reported sleep problems, but not actigraphy measures. |
| **Bitsika et al. (2016) (30)** | a, c / **L** | Australia | 150 (100%) | 6-18  11.2 (3.33) | No | Yes | No | ADOS | ABC (Community) | Child and Adolescent Symptom Inventory subscale for Generalized Anxiety Disorder (CASI-GAD) | Which Aspects of Challenging Behaviour Are Associated with Anxiety across two Age Groups of Young Males with an Autism Spectrum Disorder? | To explore the associations between IQ, age, challenging behavior, and anxiety within a sample of children with autism spectrum disorder. | MANOVA, Spearman correlation coefficients, and hierarchical regression. | Irritability negatively associated with age, and positively with anxiety. |
| **Bitsika et al. (2017) (31)** | a, c / **L** | Australia | 150 (100%) | 6-18  11.2 (3.33) | No | Yes | No | ADOS | ABC (Community) | Child and Adolescent Symptom Inventory (CASI-4) | How is Challenging Behaviour Associated with Depression in Boys with an Autism Spectrum Disorder? | To examine the association between aberrant behavior and depression in a sample of males with autism spectrum disorder. | MANOVA, Spearman correlation coefficients, and hierarchical regression. | Irritability negatively associated with age, and positively with depression symptoms. |
| **Brenner et al. (2018) (35)** | a / **L** | US | 350 (79%) | 4-21  12.9 (3.30) | No | Yes | No | SCQ, ADOS-2 | ABC (Community) | Demographic and Medical Intake Form developed by research team | Behavioral Symptoms of Reported Abuse in Children and Adolescents with Autism Spectrum Disorder in Inpatient Settings. | To examine how behavioral manifestations of trauma due to abuse are expressed in youth with autism spectrum disorder. | ANCOVA. | Irritability positively associated with history of abuse. |
| **Carpenter et al. (2022) (41)** | b / **L** | US | 185 (81%) | 3-7  4.8 (1.20) | No | No | No | ADOS-2, ADI-R | ABC (Parent/Caregiver) | VABS | Adaptive Behavior in Young Autistic Children: Associations with Irritability and ADHD Symptoms. | To examine relationships between irritability, ADHD symptoms, and adaptive behavior in autistic children. | Kolmogorov-Smirnov (K-S) test and Spearman partial correlations. | Irritability positively associated with social adaptive behaviors. |
| **Carter Leno et al. (2021) (27)** | a, b, c, d, g / **L** | UK | 52 (63%) | 13-17  15.4 (1.10) | No | No | No | ADOS-2, ADI-R | ARI / ABC (Parent/Caregiver) | Child and Adolescent Psychiatric Assessment- parent version (CAPA) | Behavioural and physiological response to frustration in autistic youth: associations with irritability. | To test the association between objectively measured response to frustration and irritability in a population-based sample of autistic youth. | Bivariate correlations, Multilevel mixed-effect models, and Negative binomial mode. | Irritability not significantly correlated with autism features, age, sex, or IQ, but positively correlated with ODD symptoms, and a flatter slope of heart rate. |
| **Chaidez et al. (2014) (61)** | f / **L** | US | 499 (86%) | 2-5 | No | Yes | Yes: Maternal education | ADOS, ADI-R, SCQ | ABC (Parent/Caregiver) | Gastrointestinal History questionnaire developed by research team | Gastrointestinal problems in children with autism, developmental delays or typical development. | To compare gastrointestinal problems among children with autism spectrum disorder, developmental delay, and typical development and examine the relationship between gastrointestinal symptoms and maladaptive behaviors. | Wilcoxon rank sum test or t-tests. | Irritability positively associated with gastrointestinal symptoms. |
| **Curran et al. (2007) (63)** | f / **S** | US | 30 (80%) | 2-18  7.4 (3.30) | No | No | No | - | ABC (Parent/Caregiver) |  | Behaviors Associated With Fever in Children With Autism Spectrum Disorders. | To investigate the effect of illness on behaviors of children with autism spectrum disorders | ANOVA. | Irritability not associated with fever. |
| **Dellapiazza et al. (2020) (39)** | b, d / **L** | France | 197 (83%) | 3-10  5.7 (2.20) | No | No | Yes: Parental education | ADOS-2, ADI-R | ABC (Parent/Caregiver) | Long‐form Sensory Profile, WISC-4, WISC-5, WPPSI-R, Kaufman Assessment Battery for Children (KABC), Brief Early Childhood Screening Assessment (BECS), Psychoeducational Profile (PEP-R), Brunet-Lezine scales | The Impact of Atypical Sensory Processing on Adaptive Functioning and Maladaptive Behaviors in Autism Spectrum Disorder During Childhood: Results From the ELENA Cohort. | To determine the prevalence and type of atypical sensory processing in children with ASD and investigate its impact on their adaptive functioning and maladaptive behaviors. | Student's T-test, the Mann-Whitney test and ANCOVA. | Irritability positively associated with sensory differences, and negatively with IQ. |
| **Dellapiazza et al. (2022) (38)** | b / **L** | France | 51 (85%) | 3-10  5.5 (2.10) | No | No | Yes: Parental education | ADOS-2 | ABC (Parent/Caregiver) | Sensory Profile | A longitudinal exploratory study of changes in sensory processing in children with ASD from the ELENA cohort. | To investigate the course of Sensory Processing among children with ASD and identify clinical variables associated with changes. | Longitudinal data analysis, time differences (Delta), and ANOVA or Kruskal Wallis tests. | Irritability positively associated with sensory differences. |
| **Estes et al. (2007) (48)** | d / **L** | US | 74 (82%) | 6  6.1 (0.23) | No | Yes | Yes: Maternal age and education | ADI-R, ADOS-G | ABC (Parent/Caregiver) | Differential Abilities Scales, VABS | Level of Intellectual Functioning Predicts Patterns of Associated Symptoms in School-Age Children With Autism Spectrum Disorder. | To investigate the relation between level of intellectual functioning and risk for associated symptoms in children with ASD. | ANOVA. | Irritability positively associated with non-verbal IQ and communication abilities. |
| **Ferguson et al. (2016) (59)** | f / **L** | US | 120 (90%) | 6-18  11.8 (3.80) | No | Yes | No | ADOS | ABC (Parent/Caregiver) |  | Associations between cytokines, endocrine stress response, and gastrointestinal symptoms in autism spectrum disorder. | To examine whether gastrointestinal symptoms in ASD were associated with increases in cortisol, a stress-associated endocrine marker, and cytokines in response to stress. | Pearson partial correlations. | Irritability negatively associated with cytokines. |
| **Ferguson et al. (2017) (60)** | f / **L** | US | 120 (90%) | 6-18  11.8 (3.80) | No | Yes | No | ADOS | ABC (Parent/Caregiver) | Questionnaire on Pediatric Gastrointestinal Disorders Rome III (QPGS Rome III) | Psychophysiological Associations with Gastrointestinal Symptomatology in Autism Spectrum Disorder. | To examine the relationship between gastrointestinal symptomatology, examining upper and lower gastrointestinal tract symptomatology separately, and autonomic nervous system functioning, as assessed by heart rate variability and skin conductance level, in a sample with ASD.  To examine relationships with co-occurring medical and psychiatric symptoms. | Pearson partial correlations, ANOVA, and Cohen's d. | Irritability positively associated with gastrointestinal symptoms. |
| **Flowers et al. (2020) (25)** | a, b, c, d / **L** | US | 145 (79%) | 9-21  16.0 (3.26) | No | No | No |  | ABC (Teacher/Educator; An adult familiar with client) | Sensory subscale on the Autism Spectrum Rating System (ASRS), Behavior Problems Inventory-  Short Form (BPI-S), VABS-2 | Associated Factors of Self-injury Among Adolescents with Autism Spectrum Disorder in a Community and Residential Treatment Setting. | To examine factors related to self-injurious behavior in an analysis of client records of children with autism in a comprehensive care center. | Spearman's correlation and regression model. | Irritability positively correlated with sensory differences, stereotypies, self-injurious behaviors, and aggression, but not with age, sex, adaptive behavior/skills. |
| **Fok et al. (2019) (46)** | c, d / **L** | US | 1,937 (87%) | 6-18  10.1 (2.96) | No | Yes | Yes: Maternal education | ADOS | ABC (Parent/Caregiver) | CBCL 6-18 | Differences in profiles of emotional behavioral problems across instruments in verbal versus minimally verbal children with autism spectrum disorder. | To explore the relationship between emotional and behavioral problems and verbal abilities by comparing profiles from two measures commonly used to assess emotional and behavioral problems in studies of children with ASD: CBCL and ABC. | Pearson correlations and ANOVA. | Irritability positively associated with externalizing and internalizing behaviours and negatively associated with verbal ability, but not after controlling for non-verbal IQ. |
| **Frazier et al. (2013) (24)** | a / **L** | US | 2,418 (87%) | 4-18  F: 9.3 (3.76), M: 9.0 (3.56) | No | Yes | Yes: Highest parental education and family income | ADOS, ADI-R, SRS, RBS-R | ABC (not specified) | Differential Ability Scales, WASI, WISC-4 | Behavioral and Cognitive Characteristics of Females and Males With Autism in the Simons Simplex Collection. | To examine differences in behavioral symptoms and cognitive functioning between males and females with ASD. | T-tests or Chi-square and multiple comparison correlations. Moderator and mediator analysis and Multiple comparison corrections. | Irritability higher in females than males, negatively correlated with age, and not associated with IQ. |
| **Frye et al. (2017) (62)** | f / **S** | US | 87 (80%) | 6.8 (3.08) | No | No | No | ADOS, ADI-R | ABC (not specified) |  | Thyroid dysfunction in children with autism spectrum disorder is associated with folate receptor alpha autoimmune disorder. | To measure blocking and binding folate receptor alpha antibodies and thyroid-stimulating hormone, free thyroxine, total triiodothyronine, reverse triiodothyronine, thyroid-releasing hormone and other metabolites in children with ASD, who also underwent behavior and cognition testing at two time points. | Cross-sectional analysis and Repeated-measures analysis (mixed model). | Irritability significantly associated with thyroid function. |
| **Gabriels et al. (2005) (42)** | b, d / **S** | US | 14 (71%) | 10.6 (7.00) | No | Yes | Yes: Mean SES | ADOS | ABC (Parent/Caregiver) | Leiter International Performance Test-Revised (Leiter-R), RBS-R | Repetitive behaviors in autism: relationships with associated clinical features. | To examine the relationships between repetitive behaviors and associated clinical features (i.e., cognitive and adaptive functioning levels, sleep problems, medication use, and other behavioral problems) in children with autism spectrum disorders. | Pearson correlations and partial correlations. | Irritability positively associated with non-verbal IQ. Irritability positively associated with repetitive behavior, but not after controlling for non-verbal IQ. |
| **Gotham et al. (2012) (43)** | c / **L** | US | 1,429 (86%) | 5-18  10.2 (3.08) | No | Yes | Yes: Maternal education | ADOS, ADI-R | ABC (Parent/Caregiver) | CBCL | Exploring the Relationship Between Anxiety and Insistence on Sameness in Autism Spectrum Disorders. | To examine the anxiety and insistence on sameness association, controlling for age and verbal IQ, assessing these target variables relationships with ASD severity and other behavioral dimensions, such as irritability and aggression. | multivariate general and linear model (GLM). | Anxiety significantly predicted irritability. |
| **Graziosi et al. (2023) (16)** | a, d / **L** | US | 457 (79%) | 4-20  13.0 (3.33) | No | Yes | Yes: Current residence and annual household income | ADOS-2 | ABC (Parent/Caregiver) | VABS-2, Leiter International Performance Scale-3 (Leiter-3) | Age and sex differences in problem behaviours in youth with autism spectrum disorder. | To examine age and sex differences in problem behaviors in youth with ASD who were diverse in age and developmental level. | Regression, Pearson's correlations, and independent-samples t-test. | Irritability negatively associated with adaptive behaviors and age, and female sex. Irritability not associated with non-verbal IQ. |
| **Griffin et al. (2022) (34)** | a, b / **S** | Australia | 75 (87%) | 7-12  7.8 (2.61) | No | No | No | ADOS-2 | ABC (Parent/Caregiver) | Short Sensory Profile-2 (SSP-2), Caregiver strain questionnaire (CGSQ) | Atypical sensory processing features in children with autism, and their relationships with maladaptive behaviors and caregiver strain. | To explore four sensory processing features; seeking, avoiding, sensitivity, and registration, and their relationships with maladaptive behaviors in children with autism, as well as with caregiver strain. | Pearson's correlations and hierarchical regression analysis. | Irritability positively associated with sensory differences and caregiver strain. |
| **Gundogdu et al. (2023) (40)** | b / **L** | Turkey | 46 (83%) | 3-9  5.3 (2.17) | No | No | Yes: SES and parental education | CARS | ABC (Parent/Caregiver) | Dunn Sensory Profile, the Turkish version | Sensory profiles, behavioral problems, and auditory findings in children with autism spectrum disorder. | To examine the relationship between sensory processing differences and behavioral problems in children with ASD. | Correlation, linear regression, and analysis of scales. | Irritability positively associated with sensory differences, autism features, and Otoacoustic Emission test failure. |
| **Hartley-McAndrew et al. (2010) (64)** | f / **S** | US | 21 (81%) | 7-23  9.5 | No | No | No | CARS, ADOS | ABC (Parent/Caregiver) |  | Autism Spectrum Disorder: Correlation between aberrant behaviors, EEG abnormalities and seizures. | To inquire whether epileptiform activity and seizures are associated with adverse behavioral manifestations in ASD population | ANOVA and ANCOVA. | Irritability not significant correlated with seizures. |
| **Henry et al. (2014) (33)** | a / **S** | US | 123 (83%) | 2-18 | No | No | No | DSM-IV-TR | ABC (Other: not specified) |  | Low Rates of Depressed Mood and Depression Diagnoses in a Clinic Review of Children and Adolescents with Autistic Disorder. | To report on the prevalence of depression diagnoses and related clinical data in an outpatient setting of children and adolescents with a diagnosis of autistic disorder. | Chi-square test. | Irritability positively associated with family history of mood disorders. |
| **Hirota et al. (2020) (66)** | h / **L** | US | 2,612 (88%) | 4-18 | No | No | No | ADOS, ADI-R | ABC (Parent/Caregiver) |  | The Network Structure of Irritability and Aggression in Individuals with Autism Spectrum Disorder. | To conduct a network analysis which conceptualizes mental health difficulties as a complex network of directly associated symptoms in individuals diagnosed with ASD. To investigate the network structure of irritability and aggression to identify bridge symptoms that link irritability and aggression and other symptom domains. | Network analysis, Walktrap communities, expected influence, and betweenness. | Irritability links aggressive behaviours to other psychopathological symptoms in the network. |
| **Johnson et al. (2018) (54)** | f / **L** | US | 177 (88%) | 3-7  4.7 (1.14) | No | Yes | Yes: Maternal education | ADOS, ADI-R | ABC (Parent/Caregiver) | CSHQ | Exploring Sleep Quality of Young Children with Autism Spectrum Disorder and Disruptive Behaviors. | To examine the association of age and IQ in Sleep disturbances in ASD. To test whether children with poor Sleep have greater daytime behavioral problems than those with better sleep. To examine whether parental stress is higher in children with greater disruptive behaviors and Sleep disturbances. | T-test. | Irritability significantly positively associated with sleep problems. |
| **Kalvin et al. (2021) (9)** | a, b, c, d / **L** | US | 81 (78%) | 8-16  G1: 12.2 (1.75), G2: 12.7 (2.06), G3: 12.5 (2.19) | No | Yes | No | ADOS-2, ADI-R | ARI (Parent/Caregiver) | Multidimensional Anxiety Scale for Children, 2nd edition (MASC-2), Home Situations Questionnaire (HSQ), DAS-2, WASI | Assessing Irritability in Children with Autism Spectrum Disorder Using the Affective Reactivity Index. | To examine ARI parent ratings in children with ASD and contributions of parent-rated anxiety and noncompliance to irritability measured by the ARI. | ANOVA, chi-square, Pearson correlation, Fisher r-to-z transformation, and hierarchical linear regression. | Irritability positively associated with anxiety, noncompliance, and repetitive and restricted behavior. No association with IQ, age, and sex. |
| **Kryza-Lacombe et al. (2020) (52)** | e / **L** | US | 47 (83%) | 8.3-19.2  13.9 (2.35) | No | No | No | ADOS, ADI-R | CBCL (Parent/Caregiver) |  | Face Emotion Processing in Pediatric Irritability: Neural Mechanisms in a Sample Enriched for Irritability With Autism Spectrum Disorder. | To identify the neural mechanisms of face emotion processing, in a sample enriched for irritability by including youth with high-functioning autism spectrum disorder. | Partial correlation, ANOVA. | Irritability levels negatively associated with activation in the left middle frontal gyrus and left inferior frontal gyrus in response to both fearful and happy faces, as well as altered connectivity between the right amygdala and left superior frontal gyrus. |
| **Lee et al. (2023) (50)** | d / **L** | Taiwan | 345 (79%) | 6-18  14.3 (2.70) | No | No | Yes: Parental education | ADI-R, SRS | ARI (Parent/Caregiver; Self) | Cambridge Neuropsychological  Test Automated Battery (CANTAB) | Autistic Symptoms, Irritability, and Executive Dysfunctions: Symptom Dynamics from Mult-Network Models. | To investigate the dynamics between autistic and irritability symptoms and executive functions in a sample of children and adolescents with ASD, their unaffected siblings, and neurotypical peers. | Cross-sectional network models. | Irritability not significantly associated with executive dysfunctions. |
| **Lundwall et al. (2017) (53)** | e / **S** | US | 45 (100%) | 3-13 | No | Yes | No | ADOS, ADI-R | ABC (Parent/Caregiver) |  | Relationship between brain stem volume and aggression in children diagnosed with autism spectrum disorder. | To determine the nature of relationships between brain structure volumes and parent-reported symptoms of aggression in children with ASD. | Logistic regression. | Irritability negatively associated with brainstem volume. |
| **Martinez-Gonzalez et al. (2021) (37)** | a / **L** | Spain | 62 (81 %) | G1: 10.9 (5.46), G2: 12.4 (7.75) | No | No | No | SCQ | Emotional state (Parent/Caregiver; relative or professional) |  | Differences in emotional state and autistic symptoms before and during confinement due to the COVID-19 pandemic. | To analyze the differences between the emotional states of a group of individuals with ASD and a neurotypical group both during and after the COVID-19 confinement. | T-test. | Irritability not statistically significantly different before and during COVID-19 confinement. |
| **Mayes et al. (2020) (28)** | a / **L** | US | 1,436 (79%) | 2-17  6.6 (3.30) | No | Yes | Yes: Parent occupation | CASD | PBS (Parent/Caregiver) |  | Sex Differences in Externalizing and Internalizing Symptoms in ADHD, Autism, and General Population Samples. | To compare sex differences in externalizing and internalizing symptoms between ADHD-Combined, ADHD-Inattentive, autism, and general population samples. | Independent t-tests and Cohen's d. | Irritability not significantly different between females and males. |
| **Mayes et al. (2022) (47)** | d / **S** | US | 1,436 (79%) | 2-17  6.6 (3.30) | No | Yes | Yes: Parent occupation | CASD | PBS (Parent/Caregiver) |  | Relationship between IQ and Internalizing and Externalizing Symptoms in Children with Autism and Children with ADHD. | To investigate the relationship between IQ and symptoms of ADHD, ODD, conduct disorder, aggression, anxiety, and depression in children with Autism and children with ADHD. To investigate the relationship between IQ and externalizing and internalizing disorders. | ANCOVA, Bonferroni post-hoc t-tests, Cohen's d. | Autistic children with IQ less than 70 had significantly less irritability. |
| **Mazurek et al. (2016) (23)** | a, c, f, h / **L** | US | 81 (86%) | 3.6-19.6  10.3 (3.80) | No | Yes | No | ADOS, ADOS-2 | C-SHARP - Hostility subscale (Parent/Caregiver) | CSHQ, Vanderbilt Attention Deficit/Hyperactivity Disorder Parent Rating Scale (VADPRS)-inattention and hyperactivity subscales | Sleep and Behavioral Problems in Children with Autism Spectrum Disorder. | To examine the relationships between specific types of sleep and behavioral problems among children with ASD. | ANOVA, Pearson's correlation, and linear regression models. | Irritability higher in females than males, but not significantly associated with race, age, bedtime resistance, sleep onset delay, or sleep disordered breathing. Irritability significantly positively correlated with physical aggression, inattention, hyperactivity, sleep anxiety, sleep duration, night wakings, daytime sleepiness, and parasomnias. |
| **Mikita et al. (2015) (44)** | c, g / **L** | UK | 47 (100%) | 10-16  12.8 (2.00) | No | No | No | ADOS-G, ADI-R, SCQ | ARI (Parent/Caregiver; Self) | The Psychosocial Stress Test (PST) | Irritability in boys with autism spectrum disorders: an investigation of physiological reactivity. | To investigate symptom reporting and mechanisms of irritability in ASD, focusing on the relation between irritability and physiological stress responses. | Pearson correlation coefficient, and ANOVA. | Higher irritability associated with dampened physiological response to stress (muted cortisol response) |
| **Molcho-Haimovich et al. (2023) (26)** | a, b, f / **H** | Israel | 237 (77%) | 1.4-8.7  4.4 (1.49) | No | Yes | No | ADOS-2 | ABC (Parent/Caregiver) | Infant/Child Sensory Profile (SP), CSHQ | Sleep disturbances are associated with irritability in ASD children with sensory sensitivities. | To examine the triadic links between sleep problems, sensory profile, and behavioral problems. | Pearson correlation coefficients, multiple regression analysis, and simple slope analysis. | Irritability positively associated with sensory differences and sleep disturbances, but not sex. |
| **Nelson et al. (2023) (65)** | h / **S** | US | 30 (90%) | 9-21  14.9 (2.99) | No | Yes | No | ADOS-2, SRS | ABC (Parent/Caregiver; Clinician) | PedsQL-4 | Health Related Quality of Life in Autistic Youth and Their Families. | To examine the quality of life in autistic individuals and their families and to evaluate associations between quality of life and measures of functioning. | Pearson correlations. | Irritability negatively associated with family functioning, daily activity, and emotional domains of PedsQL. Not associated with physical, social, cognitive, communication, worry, and family relations scores on the PedsQL. |
| **Neuhaus et al. (2019) (21)** | a, b, d / **L** | US | 2,079 (87%) | 4-18  10.3 (3.12) | No | Yes | No | ADOS, ADI-R | ABC (not specified) | VABS-2 | Linking social motivation with social skill: The role of emotion dysregulation in autism spectrum disorder. | To characterize social and emotional functioning among children and adolescents with ASD. To explore contributions of social motivation and emotion dysregulation to social skill. To consider biological sex and intellectual functioning as moderators of these associations. | Group comparison and correlations and general linear models. | Irritability significantly positively associated with social skill difficulties and female sex, and negatively with adaptive functioning. |
| **Ogur et al. (2015) (51)** | e / **S** | Turkey | 12 (100%) | 3-15 | No | No | No | M-CHAT | ABC (Parent/Caregiver) |  | Relation of behavior problems with findings of cranial diffusion tensor MRI and MR spectroscopy in autistic children. | To investigate any relation of behavior problems with cranial diffusion tensor imaging and magnetic resonance spectroscopy findings in autism spectrum disorders. | Pearson correlation test. | Irritability negatively associated with fractional anisotropy in the left frontoparietal anterior limb of the right internal capsule and left middle cerebellar peduncle. |
| **Rosen et al. (2022) (32)** | a / **L** | US | 165 (79%) | 9-18  T1: 10.0 (0.89), T2: 19.0 (1.20) | No | Yes | Yes: Caregiver education | ADOS-CSS | ABC (Parent/Caregiver; Teacher/Educator) | Demographic form | Sibling Influences on Trajectories of Maladaptive Behaviors in Autism. | To examine the impact of the presence of a sibling on developmental trajectories of teacher- and parent-reported maladaptive behaviors. To assess the influence of the sibling diagnostic profile on these trajectories among individuals with ASD or non-spectrum delays from ages 9 to 18. | Multilevel models. | Irritability not associated with family composition (siblings). |
| **Sannar et al. (2018) (57)** | f / **L** | US | 106 (76%) | 4-20  12.9 (3.40) | No | Yes | No | ADOS-2, SCQ | ABC (Parent/Caregiver) |  | Sleep Problems and Their Relationship to Maladaptive Behavior Severity in Psychiatrically Hospitalized Children with Autism Spectrum Disorder (ASD). | To examine the relationship between sleep duration and awakenings to ABC and ADOS scores in hospitalized youth with ASD and behavioral disturbance. | T-tests or Chi-square tests and linear regression analysis. | Irritability positively associated with sleep difficulties. Irritability not significantly correlated with number of minutes slept or total number of awakenings. |
| **Turkoglu et al. (2021) (36)** | a, b / **L** | Turkey | 46 (83%) | 4-17  7.9 | No | No | No | AuBC | ARI (Parent/Caregiver; Self) |  | The relationship between irritability and autism symptoms in children with ASD in COVID-19 home confinement period. | To investigate the impact of COVID-19 home confinement on ASD symptoms and irritability in children and adolescents with ASD. | Pearson correlation test and regression analyses. | Irritability significantly higher during the COVID-19 confinement period. |
| **Valicenti-McDermott et al. (2019) (55)** | f / **L** | US | 50 (94%) | 2-18  8.8 (3.00) | No | Yes | Yes: Bilingual household and maternal education | CARS | ABC (Parent/Caregiver) | Abbreviated version of CSHQ | Sleep Problems in Children With Autism and Other Developmental Disabilities: A Brief Report. | To study sleep problems in children with autism and the association with child behavioral problems in an ethnically diverse population, in a cross-sectional study with structured interview. | Chi-squared and independent t-test or nonparametrics. | Irritability not significantly associated with sleep difficulties. |
| **Viscidi et al. (2013) (22)** | d, f / **L** | US | 2,645 (87%) | 4-18  9.0 (3.60) | No | Yes | No | ADOS, ADI-R, SRS | ABC (Parent/Caregiver) |  | The association between epilepsy and autism symptoms and maladaptive behaviors in children with Autism Spectrum Disorder. | To examine the association between epilepsy and autism symptoms and associated maladaptive behaviors in children with ASD. | Poisson regression models. | Irritability significantly higher in females than males, positively associated with seizures. |
| **Williams et al. (2018) (49)** | d / **L** | US | 346 (79%) | 4-21  12.9 (3.30) | No | Yes | Yes: Household incomes and parental education | SCQ, ADOS-2 | ABC (Parent/Caregiver) |  | Problem Behaviors in Autism Spectrum Disorder: Association with Verbal Ability and Adapting/Coping Skills. | To examine the relationship between problem behaviors and verbal ability in psychiatric inpatients with ASD. | ANCOVA and hierarchical linear regressions. | Irritability positively associated with verbal ability. |
| **Yavuz-Kodat et al. (2020) (58)** | F / **L** | France | 52 (79%) | 3-10  5.4 (1.50) | No | No | No | ADOS, ADI-R | ABC (Parent/Caregiver) | CSHQ | Disturbances of Continuous Sleep and Circadian Rhythms Account for Behavioral Difficulties in Children with Autism Spectrum Disorder. | To investigate the differential impact of objectively measured sleep and circadian rhythm disturbances on behavioral difficulties in children with ASD. | Independent sample t-tests, Wilcoxon Mann-Whitney or Welch's t-tests, and dominance analysis. | Irritability positively associated with sleep difficulties. |

Domains a) demographics/ environmental, b) autism features, c) mental health, d) language/ cognition/ function, e) neurobiological, f) physical health, g) physiological, and h) multidimensional.

The risk of bias assessment: **L**) low bias, **S**) some bias, and **H**) high bias.

ABC: aberrant behavior checklist, ABI: autism behavior inventory, ADHD: attention deficit hyperactivity disorder, ADI: autism diagnostic interview, ADOS: autism diagnostic observation schedule, ANCOVA: analysis of covariance, ANOVA: analysis of variance, ARI: affective reactivity index, ASD: autism spectrum disorder, AuBC: autism behavior checklist, CARS: child autism rating scale, CASD: checklist for autism spectrum disorder, CBCL: child behavior checklist, C-SHARP: children's scale of hostility and aggression reactive/proactive, CSHQ: Children’s Sleep Habits Questionnaire, DAS: Differential Ability Scales, DSM: diagnostic and statistical manual of mental disorders, G: group, IQ: intelligence quotient, MANOVA: multivariate analysis of variance, M-CHAT: modified checklist for autism in toddlers, MR: magnetic resonance, MRI: magnetic resonance imaging, ODD: oppositional defiant disorder, PBS: pediatric behavior scale, PedsQL: pediatric quality of life inventory, RBS: repetitive behavior scale, SCQ: social communication questionnaire, SES: socioeconomic status, SRS: social responsiveness scale, T: time point, WASI: Wechsler abbreviated scale of intelligence, WISC: Wechsler intelligence scale for children, WPPSI: Wechsler preschool and primary scale of intelligence, VABS : vineland adaptive behavior scales.
